# Transfusion transmitted virus and dengue virus among healthy blood donors: A prevalence report from Jordan

**DOI:** 10.17305/bjbms.2022.7832

**Published:** 2023-05-01

**Authors:** Samer Swedan, Doaa Al-Saleh

**Affiliations:** 1 Department of Medical Laboratory Sciences, Jordan University of Science and Technology, Irbid, Jordan

**Keywords:** Transfusion transmitted virus (TTV), dengue virus (DENV), prevalence, infection, Jordan

## Abstract

Transfusion transmitted virus (TTV) is thought to contribute to non-A non-E hepatitis and other diseases. Dengue virus (DENV) is a serious mosquito-borne pathogen. Reports on TTV and DENV in Jordan and the Middle East and North Africa region are limited. Herein, the prevalence of TTV antigen and anti-DENV IgG antibodies among apparently healthy blood donors from Northern Jordan and the Northern Agwar region of Jordan was investigated using an enzyme-linked immunosorbent assay. Chi-square test and binary logistic regression were used to correlate positivity with possible infection risk factors (age, sex, residence location, and occupation). One hundred ninety apparently healthy blood donors were included in the study (age 18–54 years). TTV antigen was detected in 17.9% of the samples. Lower antigen positivity was observed among Agwar residents than non-residents (7.1% vs 24.5%; chi-square test *P* < 0.001), which was confirmed by regression analysis (odds ratio 0.262 [95% confidence interval 0.086–0.805]; *P* ═ 0.019). Antigen positivity did not differ by age, sex, or occupation. Seropositivity for anti-DENV IgG was 17.9%. Seropositivity did not differ by age, sex, or occupation. Higher seropositivity was observed among Agwar residents than non-residents (36.1% vs 9.4%; chi-square test *P* < 0.001), which was confirmed by regression analysis (odds ratio 5.420 [95% confidence interval 2.377–12.359]; *P* < 0.001). Overall, low TTV antigen prevalence and DENV seroprevalence were found among blood donors from Northern Jordan and the Northern Agwar region of Jordan.

## Introduction

Transfusion transmitted virus (TTV), also known as torque teno virus is the first identified infectious agent of the *Anelloviridae* family in humans and is suspected to be the cause of non-A to non-E hepatitis [1]. It is a small, circular, non-enveloped virus with a single-stranded DNA genome of approximately 3800 nucleotides. It was first detected in 1997 in the blood of a Japanese patient with acute post-transfusion hepatitis of unknown etiology [1, 2]. TTV has been suggested to be associated with several illnesses, including hepatitis, cancer, respiratory infections, and autoimmune diseases. However, an association between TTV and specific clinical diseases has not been established to date [1–4]. TTV exhibits a large amount of genetic diversity with several phylogenetic groups and many genotypes [2, 5].

TTV is mainly transmitted by transfusion of blood or blood components, usually from asymptomatic donors [2, 6]. However, studies have proposed other transmission routes, including respiratory, due to its presence in various biological excretions, which can potentially explain the high prevalence of TTV [3, 7]. TTV infects healthy humans of all ages. Prevalence rates differ from population to population [8], with some populations having rates up to 100% [8, 9–12].

Dengue virus (DENV) is a mosquito-borne enveloped virus, with a genome consisting of a single strand of RNA. It is responsible for dengue disease in humans. It belongs to the *Flavivirus* genus and the *Flaviviridae* family [13]. It has five antigenic serotypes (DENV-1, DENV-2, DENV-3, DENV-4, and DENV-5) [14]. The typical biological vector responsible for DENV transmission is *Aedes aegypti*, but DENV can also be transmitted by *Aedes albopictus.* The incidence of dengue disease worldwide has increased 30-fold in the past 5 decades due to several factors, including an increase in global travel, inefficient mosquito control, global warming, and increased rates of population growth [15]. To date, the *Aedes aegypti* mosquito has not been reported in Jordan, but one report documented the arrival of *Aedes albopictus* in Jordan [16]. Furthermore, both mosquito species are found in several countries surrounding Jordan, and are known to increase in spread worldwide year after year due to global warming [15].

Disease by DENV is endemic in more than 100 countries, with an estimated 400 million dengue infections occurring per year. The infection may manifest as a mild self-limiting fever (i.e., dengue fever), or as a life threating fever called dengue hemorrhagic fever/dengue shock syndrome. Dengue infection affects individuals of all age groups (infants, children, adolescents, and adults) [17].

Another source for the transmission of DENV is transfusion of blood or blood components from asymptomatic infected blood donors to recipients. The presence of non-neutralizing anti-DENV antibodies in donors’ blood may increase the risk of developing sever dengue disease in blood recipients upon infection by another DENV serotype within six months post-transfusion, a phenomenon known as antibody-dependent enhancement. Hence, DENV antibody tests are expected to be added to the panel of test performed by blood banks in endemic regions [14, 18].

Documentation of TTV prevalence worldwide may explain occurrence of diseases purported to be associated with this virus. Documentating changes in DENV prevalence worldwide is essential for establishing management strategies for health policy makers. Herein, blood samples were collected from apparently healthy blood donors from the Northern region of Jordan and from the Northern Agwar region of Jordan. Donors were selected from the Northern region of Jordan because of high elevation (more than 500 m above sea level), which less likely supports the presence of DENV vectors. Donors were selected from the Northern Agwar region since this region has a much warmer climate year-round due to very low elevation (approximately 200 m below sea level), which could support presence of DENV vectors. Samples were subjected to enzyme-linked immunosorbent assay (ELISA) for TTV antigen detection to determine the active TTV infection rate, and to ELISA for anti-DENV IgG antibodies detection to determine the prevalence of previously recovered DENV infection. Transfusion of blood containing TTV or anti-DENV IgG to blood recipients may predispose them to possible TTV-associated diseases or antibody-dependent enhancement of DENV disease. Finding high TTV or anti-DENV IgG prevalence may require changes to blood donation policies and/or screening tests in blood banks.

## Materials and methods

### Study population

Two hundred apparently healthy blood donors (age ≥ 18 years) attending the blood bank of King Abdullah University Hospital (KAUH, Irbid, Northern Jordan), the national blood bank in Irbid (Northern Jordan), Abi Obaidah Hospital (Northern Agwar), and Moa’th Bin Jabal Hospital (Northern Agwar) were included in this study using non-random accidental sampling. The inclusion and exclusion criteria were the same as for blood donation in Jordan. The inclusion criteria included both sexes, weight ≥ 50 kg for females and ≥ 55 kg for males, no donation during the past 3 months, hemoglobin levels 14–17 g/dL, and no medications in past 72 h. Exclusion criteria included having hematological disorders, not feeling well (fever, malaise, flu-like symptoms, headache, etc.), transient hypotension, and allergies (food or inhalation). Informed written consent was obtained before enrollment and a short questionnaire was completed to collect data on potential infection risk factors (age, sex, occupation, and residence location). Samples were collected from September to December 2016. All blood units were subjected to screening for hepatitis B (surface antigen and anti-core antibodies) and C (capsid antigen and anti-HCV antibodies) viruses, human immunodeficiency virus, and syphilis markers per routine work-up, and the results of the markers were obtained from the serology laboratories at each institution. Samples positive for any of the markers were excluded from the study.

### Samples

Five milliliters of venous blood were collected in plain tubes from each individual. Serum was immediately obtained by centrifugation, aliquoted in multiple 0.5 mL fractions in sterile Eppendorf tubes and stored at −80 ^∘^C for later serological analysis. New serum aliquots were used for each of the two ELISA tests.

### TTV ELISA

Testing for TTV antigen was carried out using a quantitative sandwich ELISA kit (Human TTV ELISA Kit, Neo Scientific, USA, Cat# HT0561) following the manufacturer’s instructions. ELISA wells were coated with TTV-specific antibody. Binding of the horseradish peroxidase-conjugated antibody was quantitated by measuring the absorbance of colorimetric reaction products at 450 nm. The kit included a “Standard” positive control solution to enable creation of a standard curve. ELISA plates included blank, standard, and test wells. The assay had a reported high sensitivity of 3 ng/mL and excellent specificity (per kit insert; not elaborated upon) for the detection of TTV. No significant cross-reactivity or interference between TTV and any homologous proteins was reported per kit insert.

### DENV ELISA

ELISA for anti-DENV IgG antibodies detection was carried out after the completion of TTV ELISA. New serum aliquots were tested for anti-DENV IgG using a semi-quantitative ELISA kit (anti-Dengue virus IgG Human ELISA kit, Diagnostic Automation, USA, Cat# 8116–35) following the manufacturer’s instructions. The ELISA plate wells were precoated with purified DENV virus antigens (serotypes 1–4) obtained from Vero cell cultures. Binding of antibody was detected using a peroxidase-conjugated anti-human IgG antibody followed by visualization of colorimetric reaction products at 450 nm. The kit’s reported sensitivity and specificity were 100% and 96%, respectively. The kit included negative and positive controls. Optical density values were interpreted as negative, +1 (weakly positive), +2 (moderately positive), or +3 (strongly positive). Weakly positive results might represent cross-reactivity to serum antibodies including those to other flaviviruses and were interpreted as negative for DENV IgG. Moderately and strongly positive results were interpreted as positive for DENV IgG.

### Ethical statement

This cross-sectional study was approved by the Institutional Review Board (IRB) committee of Jordan University of Science and Technology (Ref 6/98/2016).

### Statistical analysis

The Statistical Package for Social Sciences (SPSS) software version 23 (IBM, USA) was used for statistical analysis. Frequency results were compared using univariate analysis, i.e., the Chi-square test. Binary logistic regression was used to control for possible confounding factors and verify univariate analysis results. A *P* value less than 0.05 was considered statistically significant.

## Results

### Study population

Nine of the 200 samples were positive for hepatitis B core IgG and one was positive for hepatitis C virus capsid antigen, according to tests performed at the institutions that provided the samples. The other markers were negative. Hence, 10 samples were excluded and 190 samples were included in the study. The mean age of the 190 participants was 29.84 years with a standard deviation of 7.49 years and a range of 18–54 years. There were 16 females (8.4%) and 174 males (91.6%). The participants were arbitrarily divided into two age groups: 18–36 years (*n* ═ 156) and 37–54 years (*n* ═ 34). Among the participants, 56 (29.5%) resided (currently or previously) in Agwar, and 9 (4.7%) participants were military servicemen. Table 1 shows participants’ demographics by sex and age. Table S1 demonstrates all sample data and study results.

**Table 1:** Participants’ demographics by sex and age

		**Sex**		**Age group (years)**	
		**Female**	**Male**	**18–36**	**37–54**
		***n* (%)**	***n* (%)**	***n* (%)**	***n* (%)**
Age group (years)	18–36	14 (87.5)	142 (81.6)	–	–
	37–54	2 (12.5)	32 (18.4)	–	–
Agwar residence	No	15 (93.8)	119 (68.4)	103 (66.0)	31 (91.2)
	Yes	1 (6.3)	55 (31.6)	53 (34.0)	3 (8.8)
Military serviceman	No	16 (100)	165 (94.8)	148 (94.9)	33 (97.1)
	Yes	0 (0)	9 (5.2)	8 (5.1)	1 (2.9)

**Table 2:** ELISA results by sex and age

**ELISA**		**Sex**		***P* value^a^**	**Age group (years)**		***P* value^a^**
		**Female**	**Male**		**18–36**	**37–54**
		***n* (%)**	***n* (%)**		***n* (%)**	***n* (%)**	
TTV Ag	Negative	11 (68.8)	145 (83.3)	0.145	128 (82.1)	28 (82.4)	0.967
	Positive	5 (31.3)	29 (16.7)		28 (17. 9)	6 (17.6)	
Anti-DENV IgG	Negative	15 (93.8)	141 (81.0)	0.204	127 (81.3)	29 (85.3)	0.592
	Positive	1 (6.3)	33 (19.0)		29 (18.8)	5 (14.7)	

^a^Chi-square test; *P* < 0.05 is considered statistically significant. TTV Ag: Transfusion transmitted virus antigen; DENV: Dengue virus; ELISA: Enzyme-linked immunosorbent assay.

### TTV ELISA

TTV Ag was detected in sera of 34 participants, constituting 16.7% of the males (29/174) and 31.3% of the females (5/16), leading to an overall TTV Ag prevalence of 17.9%. The difference in positivity between sexes was not statistically significant (*P* ═ 0.145) (Table 2). TTV Ag positivity did not significantly differ by age (*P* ═ 0.967) or occupation (*P* ═ 0.586) (Tables 2 and 3). Significantly lower TTV Ag positivity was observed among Agwar residents (7.1%) compared to non-residents (24.5%) (*P* ═ 0.012) (Table 3). Results of the univariate analysis for each factor were confirmed by binary logistic regression after controlling for the other possible confounding factors (Table 4). Based on regression analysis, Agwar residents were at lower risk of having TTV Ag positivity than non-residents (odds ratio 0.262 [95% confidence interval 0.086–0.805]; *P* ═ 0.019) (Table 4). Among the 34 participants with TTV Ag positivity, the mean Ag concentration was 21.7 ng/mL with a standard deviation of 42.3 ng/mL, whereas the median Ag concentration was 4.0 ng/mL, and the range was 1.7–215.0 ng/mL (Table S1).

**Table 3:** ELISA results by residence location and occupation

**ELISA**		**Agwar Residence**		***P* value^a^**	**Military serviceman**		***P* value^a^**
		**No**	**Yes**		**No**	**Yes**
		***n* (%)**	***n* (%)**		***n* (%)**	***n* (%)**	
TTV Ag	Negative	104 (77.6)	52 (92.9)	0.012	148 (81.8)	8 (88.9)	0.586
	Positive	30 (24.5)	4 (7.1)		33 (18.2)	1 (11.1)	
Anti-DENV IgG	Negative	121 (90.3)	35 (62.5)	<0.001	149 (82.3)	7 (77.8)	0.729
	Positive	13 (9.7)	21 (37.5)		32 (17.7)	2 (22.2)	

^a^Chi-square test; *P* < 0.05 is considered statistically significant. TTV Ag: Transfusion transmitted virus antigen; DENV: Dengue virus; ELISA: Enzyme-linked immunosorbent assay.

**Table 4:** Binary logistic regression analyses predicting risk factors for TTV Ag and anti-DENV IgG positivity

		**TTV Ag**		**Anti-DENV IgG**	
**Variable**	**Category**	***P* value^a^**	**Adjusted OR (95% CI)**	***P* value^a^**	**Adjusted OR (95% CI)**
Age group (years)	18–36		Reference		Reference
	37–54	0.601	0.766 (0.283–2.078)	0.597	1.357 (0.437–4.353)
Sex	Male		Reference		Reference
	Female	0.376	1.686 (0.531–5.352)	0.546	0.519 (0.062–4.353)
Agwar residence	No		Reference		Reference
	Yes	0.019	0.262 (0.086–0.805)	<0.001	5.938 (2.528–13.951)
Military serviceman	No		Reference		Reference
	Yes	0.497	0.477 (0.056–4.033)	0.390	2.134 (0.379–2.018)

^a^Binary logistic regression analysis; *P* < 0.05 is considered statistically significant: Odds ratios for each independent variable were determined while adjusting for all other independent variables; TTV Ag: Transfusion transmitted virus antigen; DENV: Dengue virus; OR: Odds ratio; CI: Confidence interval.

### DENV ELISA

Out of the 190 tested sera, 156 were interpreted as seronegative for DENV (62 negative and 94 weakly positive), while 34 were interpreted as seropositive for DENV (22 moderately positive and 12 strongly positive) leading to an overall seroprevalence of 17.9%. Seroprevalence among males and females was 19.0% (33/174) and 6.3% (1/16), respectively. The difference in seroprevalence between sexes was not statistically significant (*P* ═ 0.204) (Table 2). Seroprevalence did not significantly differ by age (*P* ═ 0.592) or occupation (*P* ═ 0.729) (Tables 2 and 3). Significantly higher seroprevalence was observed among Agwar residents (37.5%) compared to non-residents (9.7%) (*P* < 0.001) (Table 3). Results of the univariate analysis for each factor were confirmed by binary logistic regression after controlling for the other possible confounding factors (Table 4). Based on regression analysis, Agwar residents were at higher risk of having DENV seropositivity than non-residents (odds ratio 5.938 [95% confidence interval 2.528–13.951]; *P* < 0.001).

## Discussion

The safety of donated blood is of paramount importance. In this study, ELISA was used to document the prevalence of current TTV and previous DENV infections via the detection of TTV antigen and anti-DENV IgG among apparently healthy blood donors. Findings of this study provide important epidemiological information regarding TTV and DENV prevalence in blood donors in Jordan, which helps to highlight differences in their prevalence across the Middle East and North Africa (MENA) region and globally. Findings could highlight the need for additional screening tests for donated blood to prevent TTV and DENV antibodies transmission to blood recipients, as TTV has been suggested to be associated with different illnesses [1–4], while DENV antibodies have been associated with antibody-dependent enhancement of DENV disease among blood recipients [14, 18].

Analysis was performed on 190 blood donor samples that were negative for hepatitis B and C viruses, human immunodeficiency virus, and syphilis infection markers. A quantitative sandwich TTV Ag ELISA kit with reported high sensitivity and excellent specificity (low cross-reactivity to homologues proteins) was utilized to document active TTV infection. Results showed a TTV prevalence of 17.9% among blood donors. The prevalence of TTV varies across regions and can reach almost 100% in certain populations [11, 12]. For example, in the MENA region, TTV prevalence among healthy individuals in Egypt was 29.0%–48.4% [19, 20], in Iran 49.3% [7], in the United Arab Emirates 75.0% [2], and in Qatar 83.4% [21]. Globally, TTV prevalence in Taiwan was 95% [9], in Brazil 6%–85% [8], and in Russia 94% [10]. Results of TTV prevalence studies vary due to several factors, including the geographical distribution of the population in the study, the diagnostic methods used, the size of the study group, and the lifestyle of the population [2, 8]. The results of our study are consistent with other studies which demonstrated that TTV infections were relatively common among healthy individuals of all ages [22]. However, TTV prevalence documented in our study was lower than in other countries in the MENA region, such as Egypt and the United Arab Emirates, which highlights potential differences in TTV prevalence even across countries within the same region and that share similar traditions and cultural norms. On the other hand, a study from 2020 using PCR for the detection of TTV among blood donors in Jordan (*n* ═ 362), found a very high prevalence of 96.1% and identified TTV genotypes 1–5 and 7 [5]. A study from 2015 utilizing PCR for detection of TTV among Jordanians residing in Qatar (*n* ═ 30) reported a prevalence rate of 90% and the identification of genotypes 3 and 5 [11]. The discordance between the findings of previous studies from Jordanians and ours may be attributed to the much higher sensitivity of the PCR technique compared to ELISA, which likely allowed detection of infection among individuals having lower viral loads. Considering the results by Sarairah et al. [5], use of TTV antigen detection by ELISA, may underreport actual TTV prevalence in the population. Hence, future studies should adopt PCR for TTV detection instead of antigen detection.

Using univariate analysis, no significant differences in TTV prevalence were observed between sexes, age groups, and occupations. These results were confirmed using binary logistic regression to control for possible confounders. Alternatively, the modest sample size and low TTV positivity may have hindered identification of differences in prevalence among the investigated factors. Interestingly, regression analysis indicated that Agwar residents had lower odds for TTV positivity than non-residents. We cannot explain the reason for this difference, but it might be attributed to population density differences between the two groups, or other unknown factors. The Agwar is an agricultural region with low population density. The samples representing the non-Agwar region were from major urban areas with much higher population densities which may facilitate TTV spread. A study from Jordan in 2020 found no differences in TTV prevalence by several attributes including sex and residence location [5]. However, this study only obtained samples from urban areas with high population densities, which may explain differences from our findings. Nonetheless, studies with larger sample sizes may be required to confirm our findings.

Our TTV investigation had several limitations. The study participants were blood donors, who in Jordan are mostly males of younger age. Hence, females and other age groups were underrepresented. A larger sample size that includes all age groups and regions in Jordan would allow for determination of accurate TTV prevalence estimates for Jordan and identification of potential factors affecting TTV prevalence among Jordanians. In our study, TTV prevalence was documented via Ag detection. The detection of TTV DNA by PCR is more sensitive than ELISA and could identify cases with low virus titers [5]. However, Ag detection was adopted because of budget limitations.

Documentating the DENV infection was done via detection of IgG antibodies. Our results showed a positive reaction for anti-DENV IgG in the serum of 17.9% of blood donors. Detection of anti-DENV IgG may indicate past or recently recovered infections, which was our goal. According to the literature [23], diagnosis of acute dengue infection should not rely only on seropositivity but also on the presence of clinical signs and symptoms of the disease. Detection of IgM antibodies which indicate a recent acute infection was not performed because of budget restriction. However, since all blood donors appeared healthy, it is likely that the seropositive individuals had previously resolved infections. Hence, the detection of IgG antibodies should be a reliable method to document DENV seroprevalence among healthy blood donors.

A systemic review for DENV seroprevalence among healthy blood donors and/or the general population from the MENA region in 2016 reported the following percentages: 7% for Saudi Arabia, 7.6% for Iran, 13.9% for Kuwait, 0.9%–16.6% for Turkey, 9.4%–27.7% for Sudan, and 28.8% for Pakistan [24]. The seropositivity for DENV in our study is in line with those across the MENA region. Reports on the incidence rate of DENV in Jordan are not available. However, a study from Jordan from 2018 with a large sample size from the general population found DENV seropositivity of 24.6% [25], a percentage that is close to what we reported. Our study focused on healthy blood donors, who do not necessarily represent the age and sex demographic distribution of Jordan. Globally, DENV seroprevalence was 15.6% for the African continent among apparently healthy individuals [26]. In Europe, seroprevalence was 14.8% [27]. In an endemic area in Mexico, DENV seroprevalence was 59% among healthy blood donors [28]. In the USA, dengue cases occurred mostly in the Southern and Southeastern regions [29]. DENV prevalence is mainly influenced by the presence of arthropod vectors, which in turn depends on a favorable climate. It was shown that DENV prevalence increases in warmer climates, which may explain, at least in part, variations in the global disease distribution [30].

Our results indicate the existence of a low, yet significant percentage of DENV seropositivity in Jordan. Nonetheless, the utilized ELISA kit has potential cross-reactivity with two flaviviruses: Japanese encephalitis virus and yellow fever virus (reported kit specificity of 96%). However, of the medically important flaviviruses, only West Nile virus (WNV) was reported in Jordan [31–33]. In a recent report, seroprevalence for WNV among Jordanians was 8.6% [32]. Based on the low prevalence of WNV and the relatively high specificity of the anti-DENV IgG ELISA, it is likely that most, if not all, moderately strong and strongly positive ELISA results represent true recovered DENV infections. A study of Jordan’s general population in 2018 reported increasing seropositivity with age [25]. However, in our study, no significant differences in DENV seropositivity were observed by age, sex, and occupation, potentially due to the modest sample size. Interestingly, participants residing in Agwar had statistically much higher odds for seropositivity than the non-residents. We hypothesize that the higher seroprevalence may be due to the year-round warm climate of the Agwar region which supports the survival of the DENV vector.

There are several limitations to the dengue investigation. The anti-DENV IgG ELISA results were not confirmed/supported by other diagnostic techniques (e.g., NS1 antigen ELISA, IgM antibodies ELISA, etc.) [23]. Using virus isolation for detection of active DENV infection is a complex and time-consuming process that demands specialized cells and experienced staff. PCR is considered the gold-standard for dengue infection diagnosis as it is sensitive, specific, and faster than virus isolation methods, but also is relatively expensive, requires specialized equipment and trained staff, and cannot document previous infections. PCR and virus isolation are considered costly for routine diagnosis in blood banks and in countries with limited financial resources. In our study, the primary focus was to identify DENV seroprevalence. Hence, documentation of active infection via virus isolation or PCR was not relevant. ELISA screening for anti-DENV antibodies is more feasible and less expensive [34]. Another limitation was the use of an ELISA kit capable of detecting four DENV serotypes (DENV-1 to DENV-4). Hence, DENV-5 could not be detected. However, since DENV-5 is a serotype that follows the sylvatic cycle in animals and rarely infects humans [35], it is unlikely to affect our overall DENV seroprevalence percentage. Also, we could not determine the prevalence of each of the serotypes, as the ELISA wells contained antigens from the four serotypes. Other limitations were the inability to rule out potential assay cross-reactivity with other flaviviruses, the small strata size for the military servicemen, and the absence of data regarding the exposure history of participants to DENV vectors.

Future studies, including a larger sample size, and encompassing various geographical locations in Jordan, in conjunction with confirmatory tests, are needed to identify/confirm variations in TTV and DENV prevalence, and potential association with population demographic factors, such as sex, age, etc.

## Conclusion

This study provides data on seropositivity for TTV antigen and anti-DENV IgG among healthy blood donors in Northern Jordan and the Northern Agwar region of Jordan. The prevalence of TTV was 17.9% and was higher among Agwar non-residents. TTV positivity did not differ by age, sex, or occupation. Currently, screening for TTV may not be recommended to ensure transfusion safety, but future investigations regarding the virulence and pathogenesis of TTV may require changes in the screening guidelines for donated blood.

The seroprevalence for DENV was 17.9% and was higher among the Agwar residents. Therefore, a percentage of blood recipients in Jordan is at potential risk of acquiring heterotypic anti-DENV IgG through blood transfusion. This preliminary finding might be useful for health policy makers, especially if global warming continues to increase the spread of the DENV vector and disease to new regions.

## Supplemental Data

Supplementary material can be found at this link: https://www.bjbms.org/ojs/index.php/bjbms/article/view/7832/2662.
